# Daily Reports on Phage-Host Interactions

**DOI:** 10.3389/fmicb.2022.946070

**Published:** 2022-07-14

**Authors:** Kamil Albrycht, Adam A. Rynkiewicz, Michal Harasymczuk, Jakub Barylski, Andrzej Zielezinski

**Affiliations:** ^1^Department of Computational Biology, Faculty of Biology, Adam Mickiewicz University, Poznań, Poland; ^2^Department of Traumatology, Orthopaedics and Hand Surgery, University of Medical Sciences, Poznan, Poland; ^3^Department of Molecular Virology, Faculty of Biology, Adam Mickiewicz University, Poznań, Poland

**Keywords:** phage, host, bacteria, archaea, phage-host interactions, database, web application

## Abstract

Understanding phage-host relationships is crucial for the study of virus biology and the application of phages in biotechnology and medicine. However, information concerning the range of hosts for bacterial and archaeal viruses is scattered across numerous databases and is difficult to obtain. Therefore, here we present PHD (Phage & Host Daily), a web application that offers a comprehensive, up-to-date catalog of known phage-host associations that allows users to select viruses targeting specific bacterial and archaeal taxa of interest. Our service combines the latest information on virus-host interactions from seven source databases with current taxonomic classification retrieved directly from the groups and institutions responsible for its maintenance. The web application also provides summary statistics on host and virus diversity, their pairwise interactions, and the host range of deposited phages. PHD is updated daily and available at http://phdaily.info or http://combio.pl/phdaily.

## Introduction

Phages play a pivotal role in many ecosystems by shaping the structure of bacterial communities (Dion et al., 2020). They are also the main drivers of horizontal gene transfer and bacterial evolution (Breitbart et al., 2018). As most viruses have narrow host ranges that span no more than a species or genus (Paez-Espino et al., 2016), they can be used to control the population of certain bacterial species with minimal risk of disturbing the entire microbiota. Thus, phages have been used in diagnostics (Schofield et al., 2012), drug design (Nixon et al., 2014), the treatment of human and animal infections (Dedrick et al., 2019; Eskenazi et al., 2022), agriculture (Buttimer et al., 2017), food preservation (Sulakvelidze, 2013), and wastewater treatment (Jassim et al., 2016).

Paradoxically, although information on host specificity is a crucial part of phage biology and a prerequisite for its practical application, it is not readily accessible. Theoretically, the databases of the National Center for Biotechnology Information (NCBI) such as RefSeq (O’Leary et al., 2016) or GenBank (Sayers et al., 2019) provide host information for most viral genomic sequences. Unfortunately, this information is stored in error-prone textual form with no direct links to the valid taxonomic classification of the host. Thus, the information is often ambiguous (e.g., simply “endosymbiont”), too generic, (e.g., “Bacteria”, “Proteobacteria”), taxonomically outdated, (e.g., “*Bacillus megaterium*” instead of *Priestia megaterium*) or misspelled (e.g., “*Bacilluls*” instead of *Bacillus*). These issues have been addressed in two excellent databases, Virus-Host DB (Mihara et al., 2016) and NCBI Virus (Hatcher et al., 2017), both of which provide access to host taxonomy based on the curation of plain-text host descriptors in GenBank and RefSeq. However, these databases only partially overlap in assignments between viral and prokaryotic species due to different genome selection criteria and host information-extraction methods (e.g., Virus-Host DB contains only viruses with complete genomes and provides host information based on a manual literature survey). Host information is also sporadically available in virus protein records from UniProt-SwissProt (Bateman et al., 2021) and annotations of protein-protein interactions from IntAct Molecular Interaction Database (Orchard et al., 2014). The MVP database (Microbe Versus Phage) provides phage–host interactions from RefSeq and GenBank with the addition of prophage sequence predictions from assembled metagenomic sequences (Gao et al., 2018). Consequently, information regarding known phage-host interactions is scattered across multiple databases, each with different content, data access, and update times. Such a situation is inconvenient for researchers and hinders attempts at systematic, statistical analyzes of phage-host interactions.

To address this problem, we have developed PHD (Phage & Host Daily), a daily updated web application that combines information on phage-host interactions from seven sources — NCBI Virus, Virus-Host DB, MVP, RefSeq, GenBank, UniProt, and IntAct. PHD provides information on hosts for prokaryotic viruses at the species level using two alternative taxonomic classification systems, NCBI Taxonomy (Schoch et al., 2020) and Genome Taxonomy Database (GTDB) (Parks et al., 2020, 2022). Virus species are classified according to NCBI Taxonomy and the International Committee on Taxonomy of Viruses (ICTV) (Gorbalenya et al., 2020; Krupovic et al., 2021). PHD also points to genome assemblies available for each virus species by keeping track of the NCBI Assembly resource (Kitts et al., 2016) and the INPHARED database of complete phage genomes (Cook et al., 2021). PHD also publishes daily reports on the current catalog of phage-host interactions. Finally, the web application offers easy access to data by providing user-friendly search, browse, and filter utilities not included in earlier phage-host databases.

## Materials and Methods

The workflow of data collection related to virus genomic sequences, host information, and taxonomic classification is shown in Supplementary Figure 1.

### Virus Sequence Data

Virus genome assemblies from GenBank and RefSeq are downloaded from NCBI (Kitts et al., 2016) using genome_updater v. 0.5.1 software^1^. The information on the assembly level of each genome (Complete Genome/Chromosome, Scaffold, Contig) is extracted from assembly report files. Nucleotide sequences of viruses present in GenBank or RefSeq but not in the Assembly database are retrieved in FASTA and flat-file formats from NCBI Virus (Hatcher et al., 2017) and the RefSeq FTP server. The obtained sequences are assigned as a “Complete Genome” if they were included in the monthly-update of complete phage genomes in INPHARED (Cook et al., 2021).

### Taxonomic Classification

National Center for Biotechnology Information (NCBI) taxonomy tables are downloaded from the NCBI FTP server. The ICTV taxonomy of viruses is retrieved from the Virus Metadata Resource at the ICTV website, and species are mapped to the corresponding NCBI taxonomy identifiers based on the RefSeq/GenBank genome accessions provided by ICTV. The GTDB taxonomy of Bacteria and Archaea is obtained from metadata files provided in the latest GTDB release. The bacterial and archaeal lineages are mapped between NCBI and GTDB taxonomies based on the NCBI taxonomy identifiers provided in the GTDB archaeal and bacterial metadata files.

### Host Information

Virus-host assignments are retrieved from: (i) the NCBI Virus website, (ii) the TSV file provided by VirusHost DB, (iii) the text files from MVP, (iv) the GenBank flat files (in the “/isolated_host = “ or “/host = “ qualifiers), (v) the protein-protein interactions from the IntAct FTP server, and (vi) the protein sequence entries in UniProt-SwissProt (“OH” line in UniProt entry). The extracted names and taxonomy identifiers of hosts are queried against NCBI Taxonomy using TaxonKit v. 0.10.1 (Shen and Ren, 2021) to retrieve complete host lineages. Only bacterial and archaeal hosts specified at the species level are included.

### Host Range

For a prokaryotic virus infecting only one host species, the host range is set to this species. For a virus infecting multiple host species, we defined the host range as the taxonomic rank of the last common ancestor of all its hosts in the NCBI taxonomic database.

### Application Development

The PHD web interface was developed in React.js (v. 17.0.2), Next.js (v. 11.1.3) and Highcharts.js (v. 10.0.0). The database querying system was developed in Django (v. 4.0.0), Django REST framework (v. 3.13.1), and Python (v. 3.9.5) using SQLite database as a management system.

## Results

### Taxonomic and Genomic Diversity of Viruses

**FIGURE 1:**
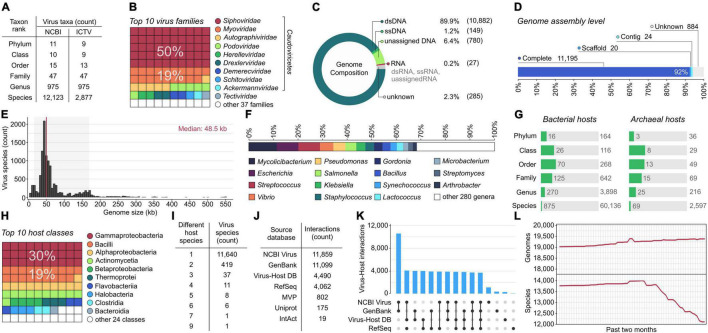
Genomic and taxonomic diversity of prokaryotic viruses and their hosts (as of May 1, 2022). **(A)** Number of different virus taxonomic units across six taxonomic ranks (from species to phylum) according to National Center for Biotechnology Information (NCBI) Taxonomy and the International Committee on Taxonomy of Viruses (ICTV). **(B)** Ten most abundant virus families represented by the highest number of virus species. **(C,D)** The number of representative viral genomes stratified by genome composition and assembly level. **(E)** Size distribution of completely sequenced virus genomes. The red vertical line indicates the median genome size, and the light gray background represents the range between the 5^th^ and 95^th^ percentiles. **(F)** Proportion of viruses isolated on the top 15 most abundant host genera (i.e., host genera infected by the highest number of viruses). **(G)** Number of different taxonomic units of bacterial and archaeal hosts across seven taxonomic ranks compared to the number of all bacterial and archaeal taxa present in NCBI Assembly. **(H)** Ten most abundant host classes represented by the highest number of known host species. **(I)** Number of virus species isolated on a different number of host species. **(J)** Comparison of the number of pairwise interactions between virus and host species in different databases. **(K)** Unique and shared virus-host interactions among four databases. The bar chart indicates the intersection size of virus-host interactions. Connected black dots on the bottom panel indicate which combination of the databases was considered for each intersection. Single, unconnected black dots represent virus-host interactions unique to each database **(L)** Number of genomes and virus species reported daily in the last 2 months (from March 1 to May 1, 2022). Virus genomes were assigned to species based on the then-most-recent NCBI Taxonomy.

As of May 1, 2022, 12,123 virus species have prokaryotic hosts reported at the species level. Only one-quarter of these viruses (24%) have been classified by ICTV, indicating a significant delay between NCBI submissions and classification by the committee. However, the number of taxa at higher ranks, from genus to phylum, is similar between NCBI and ICTV taxonomies (Figure 1A). Both systems classify prokaryotic viruses into 47 families. More than three-quarters of virus species remain in the morphotype-based *Siphoviridae, Myoviridae*, and *Podoviridae* families (Figure 1B). These umbrella groups of historical importance gather phages that are without properly resolved phylogenetic taxonomy and are scheduled for dissolution (Adriaenssens, 2021; Turner et al., 2021). Aside from these, the largest family is *Autographiviridae*, which represents 6% of the total viral species.

Consequently, most sequences in the database come from double-stranded DNA (dsDNA) viruses (Figure 1C). Single-stranded DNA (ssDNA) accounts for only 1% of viral genomes and belong to three orders: *Tubulavirales* (*n* = 71), *Petitvirales* (*n* = 67), and *Haloruvirales* (*n* = 8; two betapleolipovirus species in *Haloruvirales* have circular dsDNA genomes containing single-stranded discontinuities), and lower taxonomic units not classified at the order level (*n* = 3). RNA viruses correspond to fewer than one percent of virus species (*n* = 27) and belong to five families: *Cystoviridae* (*n* = 16), *Leviviridae* (*n* = 5), *Fiersviridae* (*n* = 4), *Steitzviridae* (*n* = 1), and *Duinviridae* (*n* = 1).

Most virus species (92%) are represented by single genome assembly. The remaining species mainly have two (4%) or three (1%) genomes assigned. The highest number of genomes have been reported for two closely related species, *Escherichia* virus G4/*Gequatrovirus G4* (*n* = 343) and *Escherichia* virus phiX174*/Sinsheimervirus phiX174* (*n* = 105). In both cases, the majority of retrieved sequences represents strains obtained during *in vitro* evolution experiments (Cuevas et al., 2009; Domingo-Calap et al., 2009). Over 92% of virus species (*n* = 11,195) have complete genomes, and the remaining viruses are represented by genomic fragments (7%; *n* = 884) or partial genomes at the contig and scaffold levels (1%; *n* = 44) (Figure 1D). Most virus species are represented only by assemblies from GenBank, but 34% are also covered by the RefSeq database.

The size of complete genomes varies between 1.4 and 551.6 kb, with no homogenous distribution (Figure 1E), which may be due to a bias linked to isolation techniques, sparse sampling of different virus taxa, or natural constraints on the size of viral genomes. Although *Campylobacter* phage C10 is the shortest phage genome sequence (1,417 bp) submitted to NCBI, the record itself (accession: MG065651) is flagged by the GenBank staff as “unverified”. The second smallest phage genome (2,435 bp) belongs to *Leuconostoc* phage L5, which is often cited as the phage with the smallest known genome (Dion et al., 2020). At the other end of the distribution (Figure 1E), there are 267 phage species (2%) with genomes of more than 200 kb, often referred to as “jumbo” or “giant” phages (Yuan and Gao, 2017; Al-Shayeb et al., 2020). Such phages have been isolated only for 73 bacterial species from 38 genera, mostly from *Erwinia*, *Vibrio*, *Aeoromans*, *Pseudomonas*, and *Klebsiella*. Phages with genomes > 500 kb (Devoto et al., 2019), *n* = 12) have been isolated from *Prevotella* species (e.g., *Prevotella* phage Lak-B8 has the largest genome of 551,627 bp).

### Virus-Host Interactions

Sequenced viruses appear to represent only a small fraction of the actual phage diversity as half of the virus species infect only eight host genera (*Mycolicibacterium*, *Escherichia*, *Streptococcus*, *Vibrio*, *Pseudomonas*, *Salmonella*, *Klebsiella*, and *Staphylococcus*) (Figure 1F). One of the reasons for such a disproportion may be biased toward culturable host taxa in isolation efforts, e.g., the SEA-PHAGES program (Science Education Alliance–Phage Hunters Advancing Genomics and Evolutionary Science) that focuses mainly on phages infecting *Mycolicibacterium smegmatis* (Jordan et al., 2014). In total, the viruses were isolated on 944 prokaryotic species including 875 bacteria and 69 archaea, accounting for 1.5% of all bacterial species (*n* = 60,136) and 2.7% archaeal species (*n* = 2,597) reported in NCBI Assembly (Figure 1G). Compared to NCBI Taxonomy, the fraction of bacterial and archaeal species with known viruses is even smaller and corresponds to only 0.2% of bacterial (*n* = 471,815) and 0.5% of archaeal species (*n* = 12,718), respectively. Although collectively, host species represent 34 classes, three-quarters of the host species fall into five classes (Gammaproteobacteria, Bacilli, Alphaproteobacteria, Actinomycetia, and Betaproteobacteria) (Figure 1H). Given that all cellular organisms are most likely prey to viral attack (Fuhrman, 1999), these gaps in host diversity indicate that phage genomic diversity and the scope of virus-host interactions remain widely uncharacterized.

To date, there are 12,725 pairwise linkages between 12,123 viral and 944 prokaryotic species. Most viruses (96.1%; *n* = 11,640) were isolated from single hosts, followed by viruses infecting two host species (3.4%; *n* = 419) (Figure 1I) mostly from the same genus or family, and sporadically with a broader host range (*Pseudomonas* virus PB1 reported in two species from different phyla, *Pseudomonas* and *Chryseobacterium*). The remaining virus species (0.5%; *n* = 64) were reported to infect more than two host species (Figure 1I). The record-holder is the *Pseudomonas* virus PRD1, known to infect nine bacteria species from the Proteobacteria phylum carrying the IncN plasmid.

Most assignments between viral and host species were retrieved from NCBI Virus (93%) and GenBank (87%), followed by Virus-Host DB (35%) and RefSeq (31%) (Figure 1J), indicating that Refseq lags behind the submission of new virus genomes (because sequence records in RefSeq additionally undergo NCBI curation). Over a quarter (29%) of the assignments were covered by all source databases (Figure 1I). Despite this overlap, these databases differ in the content of virus-host assignments. NCBI Virus provides 1,069 virus-host assignments (8%) that were not present in the other source databases. Similarly, GenBank and Virus-Host DB also have specific assignments that correspond to 3 and 2% of all interactions, respectively. The remaining source databases – RefSeq, MVP, UniProt, and IntAct – do not contain unique virus-host assignments, but provide support for 33% of the existing interactions.

### Web Interface and Data Access

Phage & Host Daily (PHD) offers two ways to access information on interactions between virus and host species: by searching for a particular virus/host taxon and browsing taxonomic trees.

The Search view allows users to look for viruses targeting bacterial or archaeal taxa of interest or prokaryotic taxa that are infected by phages from a given viral taxon. The view allows for searches corresponding to the names and identifiers used in NCBI, GTDB, and ICTV taxonomies. For convenience, the search box features an autocomplete functionality that suggests terms matching the user query. The Browse view provides a hierarchical exploration of virus-host interactions through virus or host taxonomies based on NCBI or GTDB Taxonomy. The interactive interface allows users to expand branches of virus or host trees and view the number of virus-host interactions associated with each node.

Once the query taxon is selected from either the Search or Browse view, PHD presents a table of pairwise interactions between viral and host species belonging to the query viral/host. For each virus-host interaction, PHD lists the source database(s), taxonomic affiliations for both viruses and hosts, as well as information on the virus’ genome composition and assembly completeness of the representative virus genome. This is a central component of PHD that can be filtered using multiple combinations of parameters (e.g., all virus-host interactions within *Enterobacterales* that are supported by RefSeq and Virus-Host DB and contain viruses with complete genomes).

Each virus species has an associated web page indicating host range, genomic sequences, taxonomy, and nomenclature. The available sequence data for a given virus species are organized into genome assemblies with information on assembly level, sequence length, and an indication of a representative genome, and links to NCBI Assembly and NCBI Nucleotide resources.

All virus-host interaction data and viral sequences available through the web interface can be downloaded as JSON, GenBank, and FASTA files.

## Discussion

Recent advances in metagenomics have enabled the assembly of nearly complete phage and microbial genomes from environmental samples. This has provided a unique opportunity to study the natural viral diversity and complex dynamics of phage-host interactions (Paez-Espino et al., 2016; Nayfach et al., 2021). However, metagenomically-derived phages are generally not associated with a host. This gap is slowly filled with new laboratory methods of high-throughput identification of virus-host interactions (including proximity ligation, viral tagging, phageFISH, and XRM-Seq) but these methods still require a careful interpretation by an expert and thus the paste of the discovery lags the deluge of metagenomic data (Coclet and Roux, 2021; Smith et al., 2022). These issues have prompted the development of bioinformatics tools that predict the potential host(s) based on the virus genome sequence and may select candidates for experimental verification of the interaction (Versoza and Pfeifer, 2022). Some of the most promising approaches to phage-host predictions are based on machine learning (ML) algorithms (Wang et al., 2020; Coutinho et al., 2021). As has been recently highlighted (Coclet and Roux, 2021; Versoza and Pfeifer, 2022), there is a pressing need to establish robust, comprehensive, and balanced sets suitable for training and testing ML algorithms. PHD can aid developers in constructing custom sets meeting specific criteria such as taxonomic affiliations of viruses and hosts, quality of the genome assemblies, and source databases.

The continuous mode of the PHD updates may prove useful during the current period of taxonomic upheaval. With ICTV rearranging major phage taxa to reflect their phylogenetic relations (Adriaenssens, 2021; Turner et al., 2021) and NCBI rapidly clustering sequences within the 95% identity threshold delineating species (Figure 1L), each day brings us closer to a comprehensive and well-organized classification scheme that facilitates research in all phage-related fields.

## Conclusion

Phage & Host Daily (PHD) provides a single, convenient interface that allows for rapid access to an exhaustive set of experimentally verified phage-host interactions and provides up-to-date taxonomic classifications for all phages and hosts. We hope that our service will become a convenient one-stop-shop for biologists and bioinformaticians interested in finding novel, alternative hosts of known phages, spotting the bacterial taxa that might be neglected during earlier studies, and interpreting ecological relations observed in the environment. It can also be used by developers of bioinformatic tools to compile well-annotated phage and host datasets for their tools. Finally, our data can help to uncover links between genomics and the phylogeny of prokaryotic viruses, and their host range.

## Data Availability Statement

The original contributions presented in this study are included in the article/Supplementary Material, further inquiries can be directed to the corresponding author.

## Author Contributions

AZ conceived and supervised the project. AZ and AR implemented the database and methods for data collection. KA designed and implemented the user interface. AZ and MH prepared the figure. AZ, MH, and JB analyzed the data and wrote the manuscript. All authors reviewed and approved the manuscript.

## Conflict of Interest

The authors declare that the research was conducted in the absence of any commercial or financial relationships that could be construed as a potential conflict of interest.

## Publisher’s Note

All claims expressed in this article are solely those of the authors and do not necessarily represent those of their affiliated organizations, or those of the publisher, the editors and the reviewers. Any product that may be evaluated in this article, or claim that may be made by its manufacturer, is not guaranteed or endorsed by the publisher.

## Footnotes

1
https://github.com/pirovc/genome_updater

## Supplementary Material

Supplementary Figure 1 Overview of the methods implemented in the PHD web application to collect information regarding interactions between viruses and prokaryotic host species. **(1)** Names and/or NCBI taxonomy identifiers (taxIds) of hosts are extracted from nucleotide/protein sequence records of viruses available in six source databases (NCBI Virus, RefSeq, Virus-Host DB, MVP, UniProt-SwissProt, and IntAct). **(2)** The extracted host names/taxIds are queried in TaxonKit against NCBI Taxonomy to retrieve full taxonomic lineages of hosts including their names, ranks, and taxIds. Only prokaryotic host species from Bacteria or Archaea are included in further steps. **(3)** Additional taxonomic information (if available) for each prokaryotic host species is retrieved from the Genome Taxonomy Database (GTDB). **(4)** Interaction assignments between virus sequence records and the prokaryotic host species are collected from the source databases. **(5)** Virus taxIds provided in sequence records are used to retrieve virus taxonomic lineages from NCBI Taxonomy. The obtained virus species taxIds or sequence accessions are used to retrieve virus taxonomic lineages (if available) in the International Committee on Taxonomy of Viruses (ICTV). Sequence accessions are then assigned to the appropriate virus species. For example, three genomic sequences (MN125599, MN125600, and NC_049813) belong to the *Veterinaerplatzvirus vv12210I* species. **(6)** Sequence accessions within virus species are grouped into genome assemblies based on metadata provided in the NCBI Assembly database. For example, two sequence accessions - MN125599, MN125600 - are part of one genome assembly from GenBank (assembly accession: GCA_009903655) while the third sequence NC_049813 is a separate genome assemble from RefSeq (assembly accession: GCF_009671745). Assembly level category (i.e., Complete or Scaffold or Contig or unknown) is assigned to each virus assembly based on information provided by NCBI Assembly and INPHARED databases. **(7)** Source databases are assigned to each interaction between virus and host species. For example, the interaction between *Veterinaerplatzvirus vv12210I* and *E. coli* was covered by three source databases (i.e., NCBI Virus, Virus-Host DB, and RefSeq).
