# Supplementary figures and images for: Daily Reports on Phage-Host Interactions
Source: Front Microbiol. 2022 Jul 14;13:946070. doi: 10.3389/fmicb.2022.946070 (PMC9329054; doi:10.3389/fmicb.2022.946070)

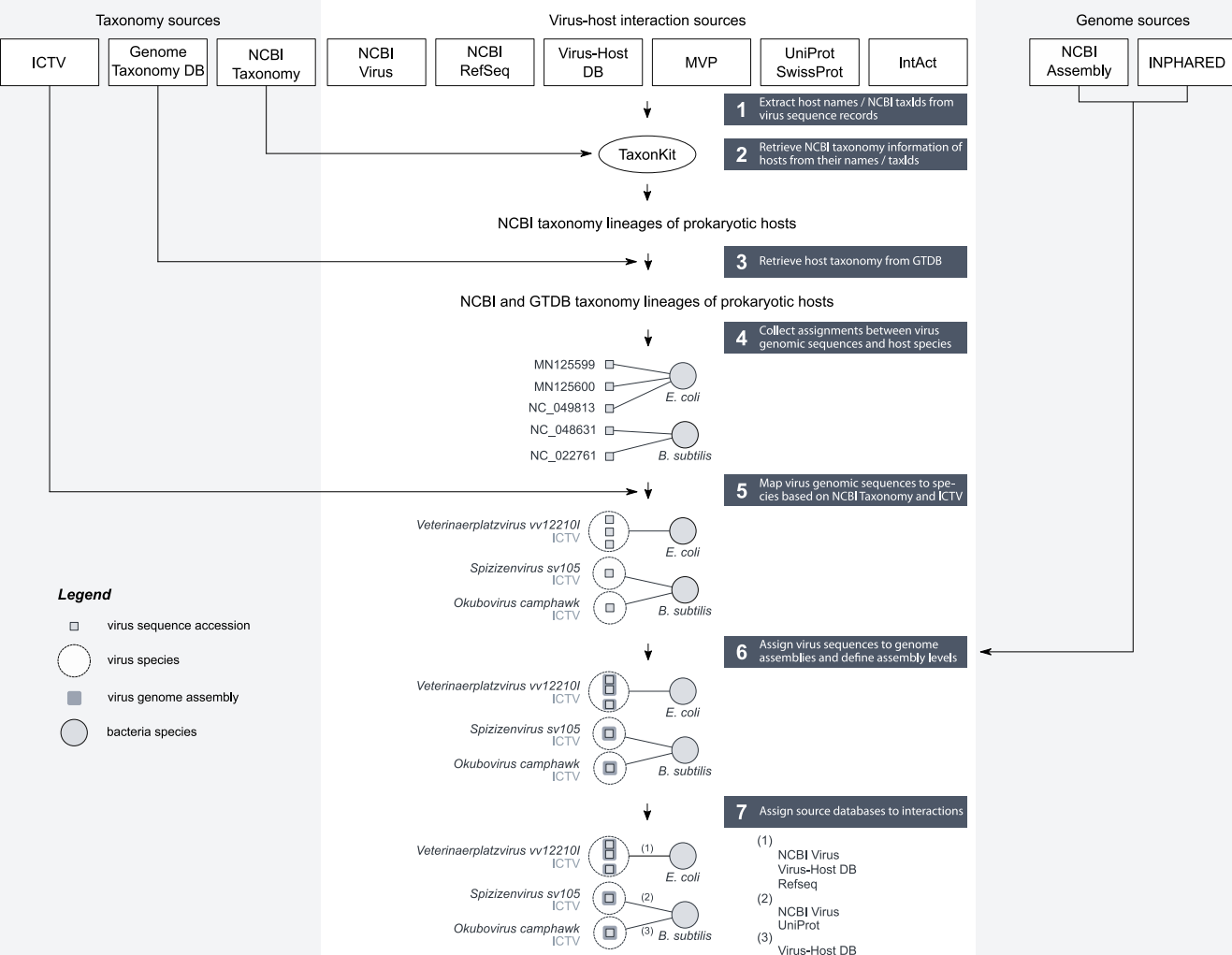

Supplement: Supplementary Figure 1 — Overview of the methods implemented in the PHD web application to collect information regarding interactions between viruses and prokaryotic host species. (1) Names and/or NCBI taxonomy identifiers (taxIds) of hosts are extracted from nucleotide/protein sequence records of viruses available in six source databases (NCBI Virus, RefSeq, Virus-Host DB, MVP, UniProt-SwissProt, and IntAct). (2) The extracted host names/taxIds are queried in TaxonKit against NCBI Taxonomy to retrieve full taxonomic lineages of hosts including their names, ranks, and taxIds. Only prokaryotic host species from Bacteria or Archaea are included in further steps. (3) Additional taxonomic information (if available) for each prokaryotic host species is retrieved from the Genome Taxonomy Database (GTDB). (4) Interaction assignments between virus sequence records and the prokaryotic host species are collected from the source databases. (5) Virus taxIds provided in sequence records are used to retrieve virus taxonomic lineages from NCBI Taxonomy. The obtained virus species taxIds or sequence accessions are used to retrieve virus taxonomic lineages (if available) in the International Committee on Taxonomy of Viruses (ICTV). Sequence accessions are then assigned to the appropriate virus species. For example, three genomic sequences (MN125599, MN125600, and NC_049813) belong to the Veterinaerplatzvirus vv12210I species. (6) Sequence accessions within virus species are grouped into genome assemblies based on metadata provided in the NCBI Assembly database. For example, two sequence accessions - MN125599, MN125600 - are part of one genome assembly from GenBank (assembly accession: GCA_009903655) while the third sequence NC_049813 is a separate genome assemble from RefSeq (assembly accession: GCF_009671745). Assembly level category (i.e., Complete or Scaffold or Contig or unknown) is assigned to each virus assembly based on information provided by NCBI Assembly and INPHARED databases. (7) Source database [file Data_Sheet_1.PDF]
